# Association between temperature, sunlight hours and alcohol consumption

**DOI:** 10.1371/journal.pone.0223312

**Published:** 2019-09-30

**Authors:** Hannes Hagström, Linnea Widman, Erik von Seth

**Affiliations:** 1 Division of Hepatology, Department of Upper GI, Karolinska University Hospital, Stockholm, Sweden; 2 Clinical Epidemiology Unit, Department of Medicine, Solna, Karolinska Institutet, Stockholm, Sweden; 3 Unit of Biostatistics, Department of Environmental Medicine, Karolinska Institutet, Stockholm, Sweden; Columbia University, UNITED STATES

## Abstract

**Background:**

Alcohol is a major risk factor for liver cirrhosis. Recently, it was proposed that colder climate might causally lead to increased consumption of alcohol.

**Methods:**

We performed an ecologic study, using monthly updated data on mean temperature, sunlight hours and alcohol consumption from ten regions in Sweden, using publicly available data. A generalised additive model, adjusted for region, was applied to examine the association between mean temperature and mean sunlight hours with mean alcohol consumption.

**Results:**

We found a non-linear inverse association between mean monthly temperature and mean alcohol consumption, suggesting that warmer temperature was associated with increased alcohol consumption and colder temperature with a decreased consumption. We found no association between mean sunlight hours and alcohol consumption. Consumption was highest during public holidays.

**Conclusions:**

We found no association between a colder climate and increased alcohol consumption. Socio-economic factors are likely to explain the suggested association.

## Introduction

A recent study examined the association between climate and alcohol consumption [1]. By linking global climate data, available statistics on annual consumption of alcohol and estimates of alcoholic cirrhosis across different countries, Ventura-Cots et al concluded that cold temperatures and few hours of sunlight may causally increase alcohol consumption. The authors further suggest that public health measures aimed at preventing excessive alcohol consumption should focus on regions with colder climates.

However, these findings seem counter-intuitive from a northern European perspective, and the research methodology and interpretations were disputed in an accompanying editorial by experienced epidemiologists [2]. Despite a colder climate and significantly less yearly hours of sunlight, mean alcohol consumption in the Nordic countries is lower compared to countries in southern Europe [3]. As stated by Ventura-Cots et al, numerous factors might influence alcohol consumption at the population level. The authors also suggest that seasonal variability within countries might be one way to avoid bias.

This motivated us to investigate if seasonal variability of temperature and sunlight hours is associated with increased alcohol consumption in Sweden.

## Methods

In this ecological study, we obtained data on mean monthly alcohol consumption (expressed here as litres of pure alcohol consumption per capita per month) from ten Swedish regions from the south to the north between 2008 and 2017 from the Swedish Council for Information on Alcohol and other Drugs (CAN, www.can.se). Publicly available data on monthly mean temperature and mean sunlight hours from these regions using calibrated pyrheliometers and thermometers from recording stations was retrieved from the Swedish Meteorological and Hydrological Institute (available at https://data.smhi.se/met/climate/time_series/month/vov_pdf/). A generalised additive model, adjusted for region, was applied to examine the association between mean temperature and mean sunlight hours with mean alcohol consumption [4]. We also stratified the data, looking separately on the four most northern regions and the six most southern regions to examine if patterns of alcohol consumption differed. Temperature and sunlight were transformed using a nonparametric spline while fitting the GAM model, using the *mgcv* package in *R*, version 3.5.2.

## Results

**Fig 1:**
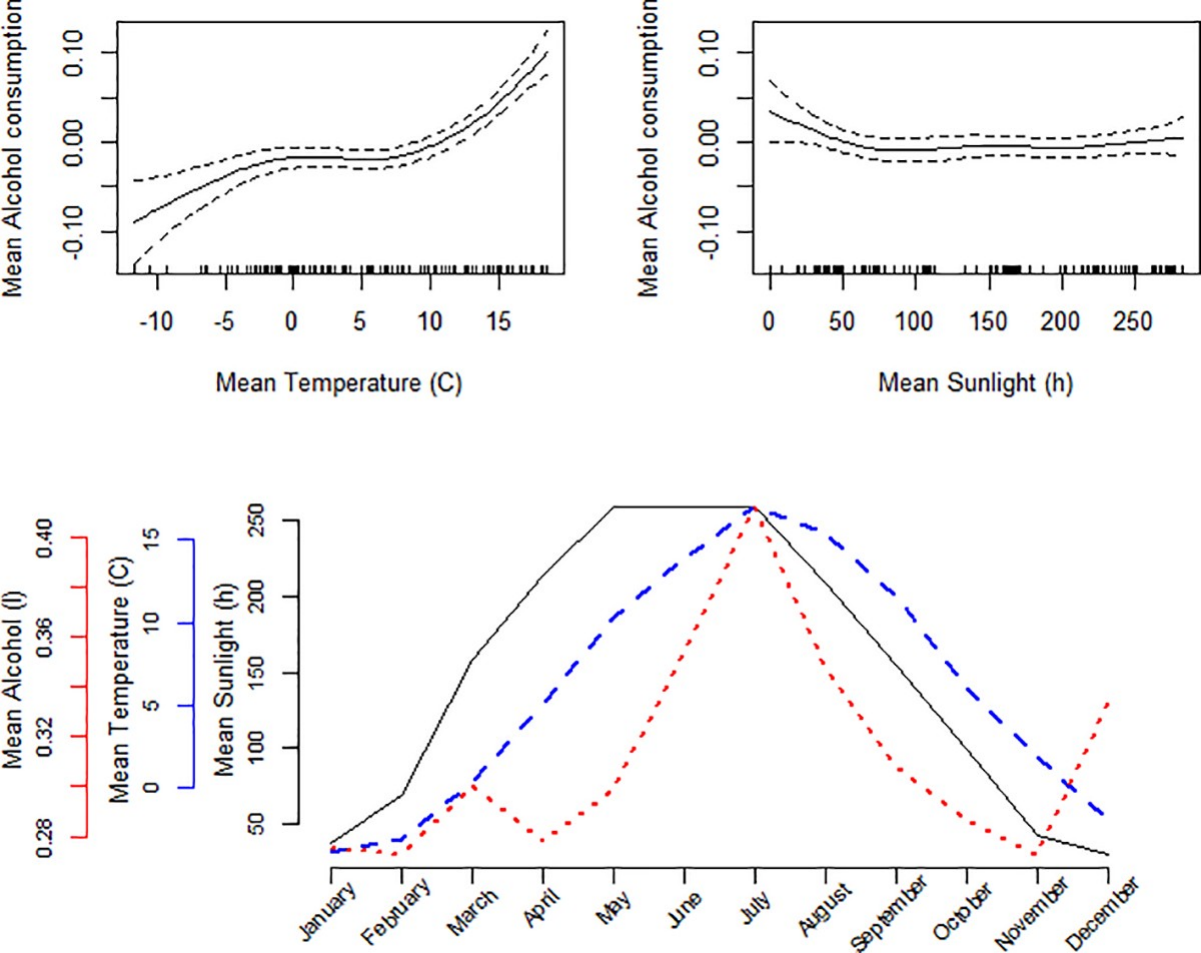
Spline plots for mean temperature (°C) and sunlight hours when fitting a generalized additive model adjusted for region on alcohol data. Line graph of mean temperature (°C), sunlight hours and alcohol consumption (litres of 100% alcohol) per month in ten Swedish regions between 2008 and 2017.

**Fig 2:**
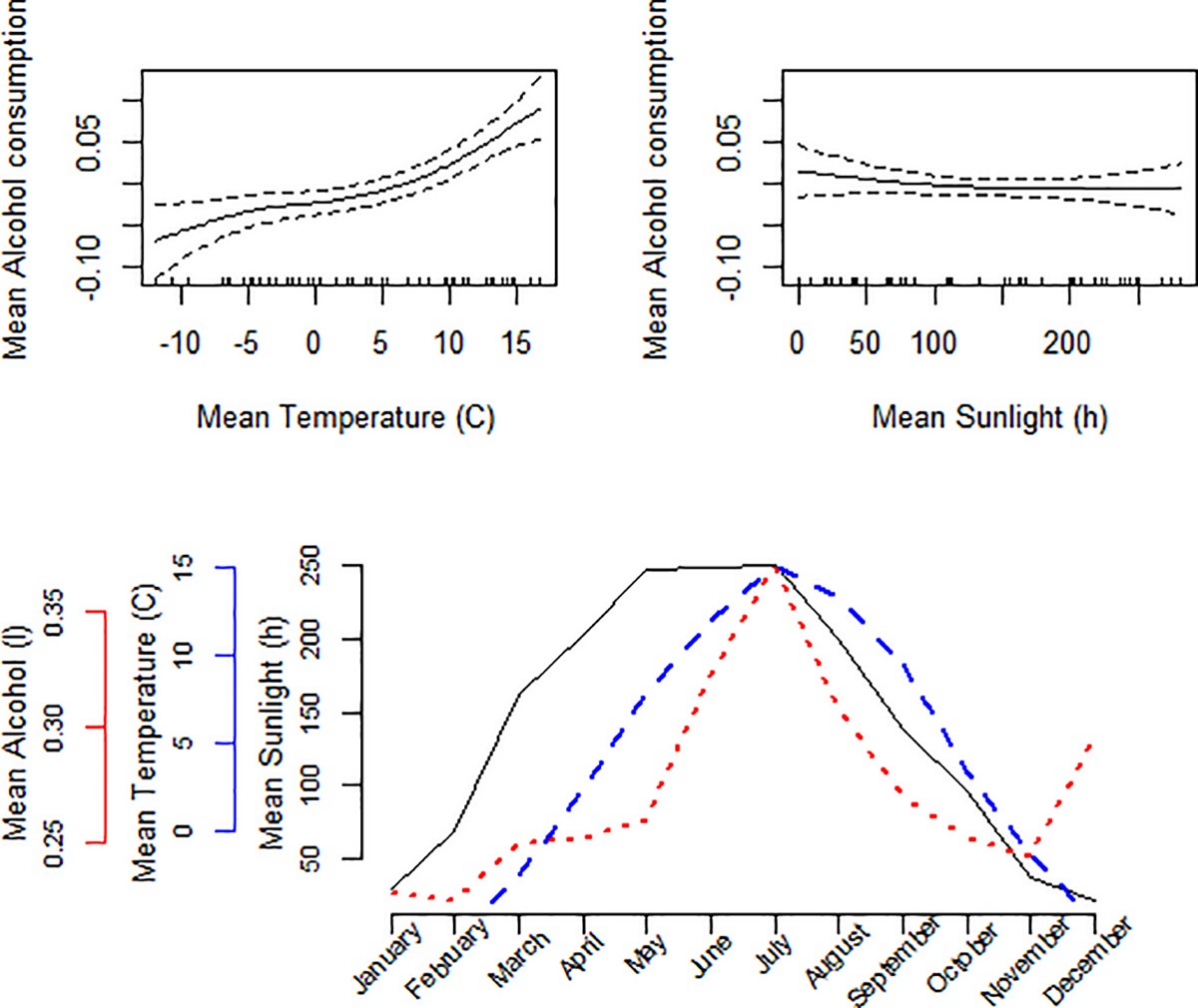
Spline plots for mean temperature (°C) and sunlight hours when fitting a generalized additive model adjusted for region on alcohol data. Line graph of mean temperature (°C), sunlight hours and alcohol consumption (litres of 100% alcohol) per month in the four most northern regions between 2008 and 2017.

**Fig 3:**
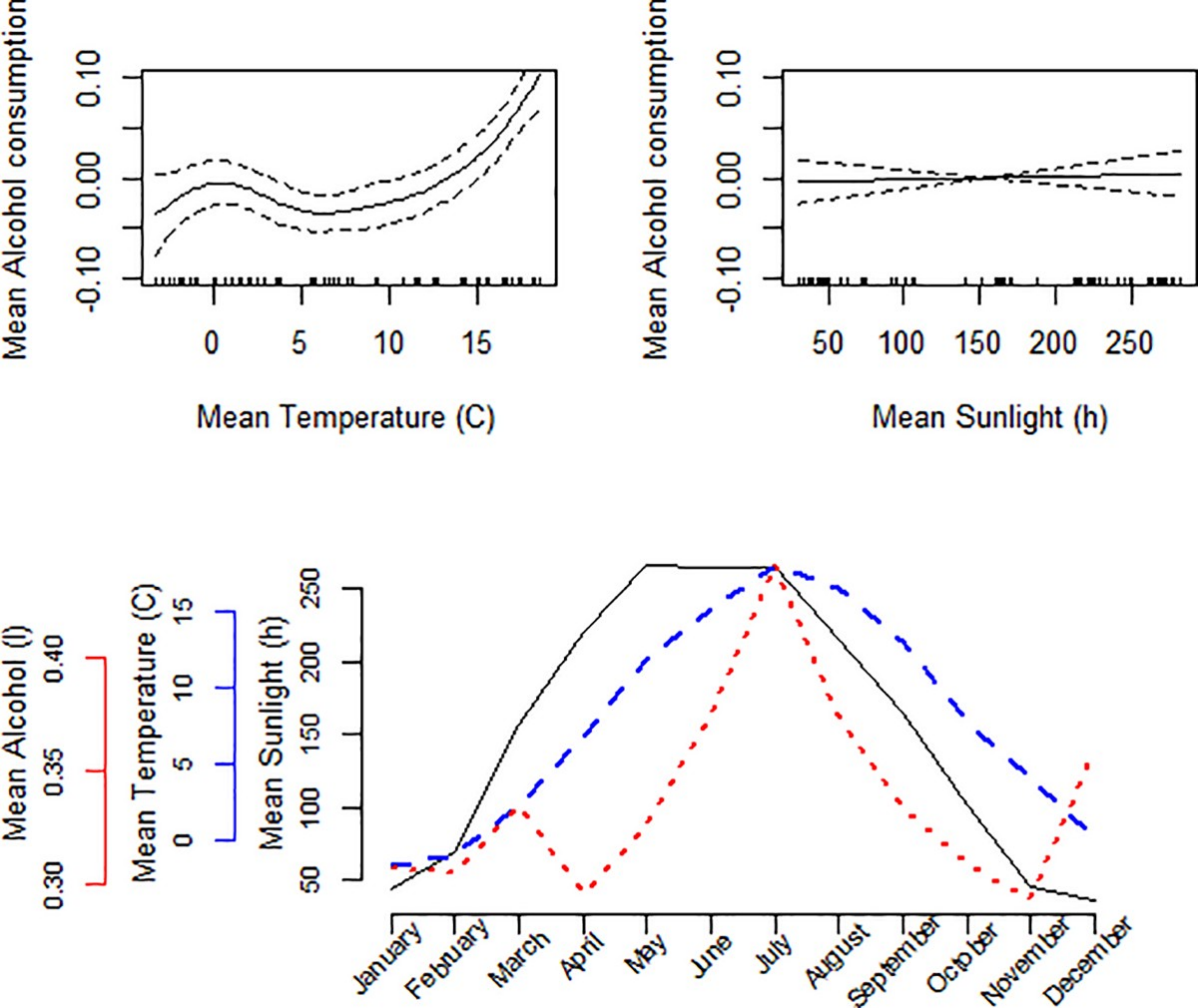
Spline plots for mean temperature (°C) and sunlight hours when fitting a generalized additive model adjusted for region on alcohol data. Line graph of mean temperature (°C), sunlight hours and alcohol consumption (litres of 100% alcohol) per month in the six most southern regions between 2008 and 2017.

Across the study period, the highest mean alcohol consumption was found in the hottest months of July (mean 0.41 litres of pure alcohol per person, mean temperature 17.0°C) and June (0.35 litres and 13.9°C). There was also a peak in December (0.33 litres and -1.9°C). The lowest consumption was found in the months of February and November (0.27 litres), which were also among the coldest months (temperature -3.1°C and +1.9°C, respectively). We found a non-linear inverse association between mean monthly temperature and mean alcohol consumption, meaning that warmer temperature was associated with increased alcohol consumption and colder temperature with a decreased consumption. However, we found no association between mean sunlight hours and alcohol consumption. These results together with the relation between monthly data on alcohol consumption, sunlight hours and temperature are presented in Fig 1. Mean alcohol consumption was lower in the four northern regions compared to the six southern regions, but the same pattern of increasing consumption during summer and in December was seen in both northern (Fig 2) and southern (Fig 3) Sweden.

## Discussion

These findings, using more detailed data on climate than in the Ventura-Cots study, go well in hand with our clinical experience of alcohol consumption in Northern Europe, where a high consumption is found during public holidays such as summer and during Christmas. Thus, we found no evidence for an association between colder and darker climate and increased alcohol consumption in Sweden, rather the opposite.

Strengths of the current study include the monthly updated data across a long period of time from several regions, credible data on mean alcohol consumption and highly accurate data on climate. Limitations include the ecologic design without individual-level data on a single measure of alcohol consumption, lack of more detailed socio-economic confounders that may account for differences in alcohol consumption patterns, and that we could only obtain data from a single country.

We infer that consumption of alcohol on a population level is more complicated than crude measures of temperature and sunlight, and that targeted interventions on alcohol policies should not be limited to periods and regions with a colder and darker climate.
